# Metal-Dilution Effect on Spin Transition Behavior of Solvated/Desolvated Hydrogen-Bonded Cobalt(II)-Organic Frameworks

**DOI:** 10.1021/acsomega.4c10686

**Published:** 2025-01-16

**Authors:** Keisuke Yamato, Takuya Kanetomo, Masaya Enomoto

**Affiliations:** Department of Chemistry, Faculty of Science Division I, Tokyo University of Science, 1-3 Kagurazaka, Shinjuku-ku, Tokyo 162-8601, Japan

## Abstract

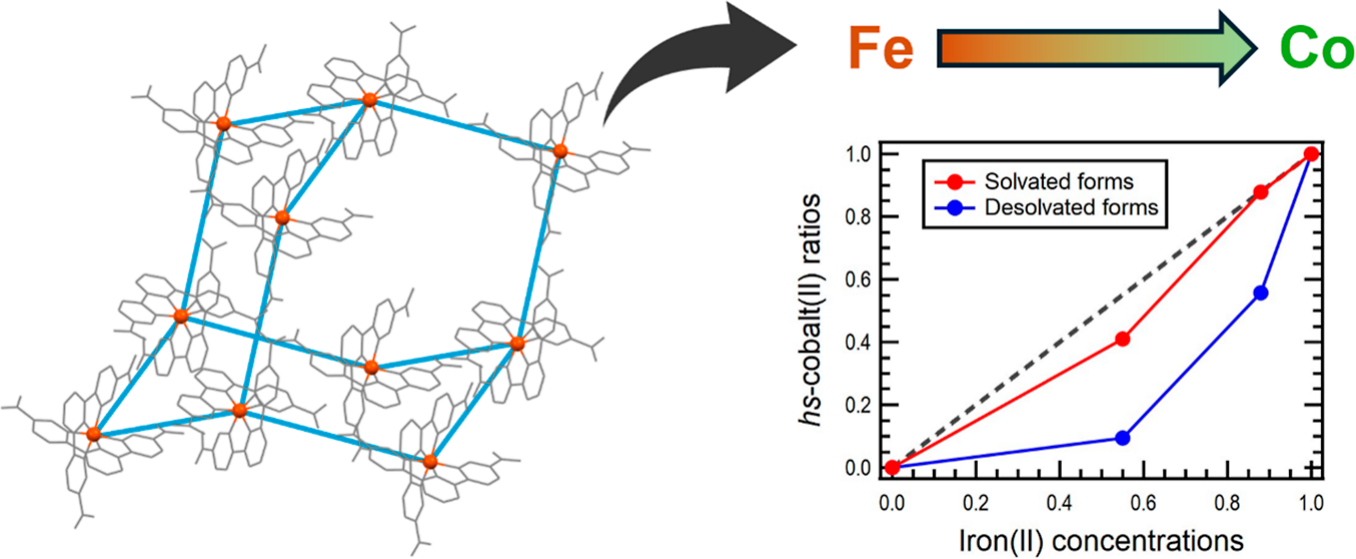

Terpyridine-based cobalt(II) complex, [Co^II^(HL)_2_] (**1**; H_2_L = 2,2′:6′,2″-terpyridine-5,5″-diyl dicarboxylic acid), forms a hydrogen-bonded diamond framework with solvent absorption and desorption capabilities. The desolvated form (**1**·desolv) exhibits spin transition (ST) behavior accompanied by thermal hysteresis. To investigate the effect of metal-dilution, an Fe^2+^ center, which has a low-spin state (*S* = 0) and coordinates to two terpyridine moieties, was introduced. The resulting complexes, [Co^II^_*x*_Fe^II^_1–*x*_(HL)_2_], where *x* = 0.88 (**2**), 0.55 (**3**), and 0 (**4**), demonstrated a significant influence of metal-dilution on the desolvated forms, but not on the solvated forms. Namely, the spin state is more strongly affected by the presence of solvent than by metal-dilution. However, in the absence of solvent, the Fe^2+^ ratio significantly impacts the ST behavior.

## Introduction

Hydrogen-bonded metal–organic frameworks (H-MOFs) use a metal complex as the building block linked by H-bonds.^1−12^ These frameworks are used in the development of molecular porous materials such as conventional MOFs^13−15^ and covalent organic frameworks.^16,17^ The degree of H-bonding influences the flexibility between the units and can be tuned by manipulating the electrostatic interactions between the donor and acceptor units.^18^ The strategic molecular design of these systems, which is dependent on intermolecular interactions, remains a challenge.

The ligand 2,2′:6′,2″-terpyridine-5,5″-diyl dicarboxylic acid (H_2_L), is capable of coordinating to the metal center via the terpyridine (tpy) moiety while simultaneously forming H-bonds through the carboxy site. In particular, it is known that the [Co^II^(tpy)_2_] complexes can exhibit spin transition (ST) between two spin states, high-spin (*hs*; *S* = 3/2) and low-spin (*ls*; *S* = 1/2) states, by external stimuli.^19−30^ This ST behavior has potential for various applications: for example, sensors, data storage, and molecular switches.^31−33^ Recently, we have reported that a novel cobalt(II) complex, [Co(HL)_2_](DMF)_1.2_(H_2_O)_2.4_ (**1**·solv), forms a diamond-like framework with Co^II^(tpy)_2_ units bridged by the H-bond between the terminal carboxy/carboxylate sites.^34^ The 4-folded diamond framework has a one-dimensional (1-D) cavity that can accommodate crystal solvents. Interestingly, **1** exhibits guest-dependent magnetic behavior. In its solvated form, it shows a simple magnetic behavior attributed to the *hs*-Co^2+^ center. On the other hand, the desolvated form exhibits ST behavior with an asymmetric thermal hysteresis loop. Namely, the spin moment initially decreases and then increases upon heating, corresponding to normal and reverse ST, respectively, while upon cooling, only normal ST behavior is observed.

Effective manipulation of the dilution concentration provides a valuable approach to control the magnetic properties in the ST materials.^35−39^ The introduction of magnetically inactive metal ions serves to separate active metal centers, thereby influencing volume elasticity and cooperativity tuning. Compound **1** exhibits guest-dependent magnetic behavior due to its flexible H-bonded framework. However, the relationship between the ST behavior and the cooperativity resulting from the H-bonded framework remains uncertain. To explore the metal-dilution effect in **1**, the Fe^II^(tpy)_2_ unit can serve as an inactive metal center, since it exhibits non-ST behavior in the *ls* state (*S* = 0).^40^ In a 6-coordinated octahedral coordination environment, the *ls*-Fe^2+^ center has an ionic radius of 0.61 Å, which is smaller than *hs*-Co^2+^ (0.745 Å) and close to *ls*-Co^2+^ centers (0.65 Å).^41^ The introduction of *ls*-Fe^2+^ with its small ionic radius could cause the lattice to shrink, and therefore it is assumed that the Co^2+^ center adopts the *ls* state with the ionic radius like that of Fe^2+^. In this study, we have prepared [Co^II^_*x*_Fe^II^_1–*x*_(HL)_2_](DMF)_*a*_(H_2_O)_*b*_, where *x* = 1 (**1**·solv; *a* = 1.2 and *b* = 2.4),^34^ 0.88 (**2**·solv; *a* = 1.1 and *b* = 2.4), 0.55 (**3**·solv; *a* = 0.9 and *b* = 2.2), and 0 (**4**·solv; *a* = 0.9 and *b* = 3.1). In the solvated form, increasing Fe^2+^ concentration resulted in a significant stabilization toward the *hs*-Co^2+^ center. On the other hand, the desolvated form showed the significant metal-dilution effect.

## Experimental Section

Ligand H_2_L was prepared according to the reported procedure.^34^ Anhydrous *N*,*N*-dimethylformamide (DMF) was purchased from KANTO Chemical Co., Inc. and used without further purification. The DMF and distilled water solvents were used with a small amount of ascorbic acid, after bubbling with Ar gas, to avoid the oxidation of the Fe^2+^ ion. Elemental analyses were performed using a PerkinElmer Series II CHNS/O 2400 analyzer. Infrared (IR) spectra were obtained on a FT/IR-4600 spectrometer (JASCO) using a diamond attenuated total reflectance (ATR) method. The spectral data are obtained by recording the major peaks in wavenumbers (cm^–1^) in a spectral window of 4000–400 cm^–1^. Thermogravimetry (TG) and differential thermal analysis (DTA) were performed on a Bruker AXS TG2010SA instrument. The temperature scan rate was 5 K min^–1^ in the range of 300–700 K. Powder X-ray diffraction (PXRD) spectra were recorded using a Rigaku MiniFlex600 diffractometer (Cu Kα radiation: λ = 1.541862 Å) in the range of 303–473 K. The Co/Fe ratio in the mixed Co–Fe crystals (**2** and **3**) was determined by inductively coupled plasma mass spectroscopy (ICP–MS) analysis using an Agilent 7850 ICP–MS and energy-dispersive X-ray spectroscopy (EDS) on a scanning electron microscope (SEM; JEOL JSM-IT800).

### Synthesis of [Fe^II^_0.12_Co^II^_0.88_(HL)_2_]·(DMF)_1.1_(H_2_O)_2.4_ (**2**·solv)

Compound H_2_L (115.8 mg, 0.361 mmol) was dissolved in anhydrous DMF solvent (14 mL), and the resulting solution was added to FeCl_2_·4H_2_O (2.70 mg, 0.0136 mmol) and CoCl_2_ (17.71 mg, 0.136 mmol) dissolved in H_2_O (4 mL). The reaction mixture was stirred for 15 min. The filtrate, passed through a cotton filter, was subjected to crystallization under 1,4-dioxane vapor diffusion for 5 days. The dark brown block crystals were collected, and the yield was 53.1 mg (0.0640 mmol, 43%). Mp. 310 °C (decomp). Anal. Calc. for C_37.3_H_32.5_Co_0.88_Fe_0.12_N_7.10_O_11.5_: C, 54.44; H, 3.99; N, 12.09%. Found: C, 54.39; H, 4.11; N, 12.34%. IR (ATR, Figure S1): 3481, 3071, 1715, 1602, 1446, 1363, 1251, 774, 720, and 682 cm^–1^. EDS (15.00 kV, Figure S2): Fe, 13.2(3); Co, 86.8(9)%. ICP–MS: ^56^Fe, 11.80; ^59^Co, 88.20%.

### Synthesis of [Fe^II^_0.45_Co^II^_0.55_(HL)_2_]·(DMF)_0.9_(H_2_O)_2.2_ (3·solv)

Compound H_2_L (144.6 mg, 0.450 mmol) was dissolved in anhydrous DMF solvent (18 mL), and the resulting solution was added to FeCl_2_·4H_2_O (12.33 mg, 0.0621 mmol) and CoCl_2_ (16.08 mg, 0.124 mmol) dissolved in H_2_O (5 mL). The reaction mixture was stirred for 15 min. The filtrate, passed through a cotton filter, was subjected to crystallization under 1,4-dioxane vapor diffusion for 7 days. The dark black block crystals were collected, and the yield was 61.5 mg (0.0742 mmol, 40%). Mp. 314 °C (decomp). Anal. Calc. for C_36.70_H_30.70_Co_0.55_Fe_0.45_N_6.90_O_11.10_: C, 54.85; H, 3.86; N, 12.03%. Found: C, 54.47; H, 4.17; N, 12.34%. IR (ATR, Figure S3): 3410, 3070, 1711, 1656, 1448, 1369, 1253, 772, 718, and 680 cm^–1^. EDS (15.00 kV, Figure S4): Fe, 47.4(6); Co, 52.6(8)%. ICP–MS: ^56^Fe, 44.72; ^59^Co, 55.28%.

### Synthesis of [Fe^II^(HL)_2_]·(DMF)_0.9_(H_2_O)_3.1_ (**4**·solv)

Compound H_2_L (60.92 mg, 0.190 mmol) and FeCl_2_·4H_2_O (6.21 mg, 0.0312 mmol) were dissolved in DMF (25 mL). The reaction mixture was stirred for 15 min, and the solution was crystallized under 1,4-dioxane vapor diffusion for 7 days. The dark purple block crystals were collected, and the yield was 14.7 mg (0.0180 mmol, 58%). Mp. 313 °C (decomp). Anal. Calc. for C_36.70_H_32.50_FeN_6.90_O_12.00_: C, 53.88; H, 4.01; N, 11.82%. Found: C, 53.79; H, 4.17; N, 12.04%. IR (ATR, Figure S5): 3406, 3066, 1705, 1646, 1449, 1368, 1249, 770, 719, and 678 cm^–1^.

### Single-Crystal X-ray Diffraction (SCXRD)

X-ray diffraction data of **4**·solv at 90 K were collected on a Bruker D8 Quest diffractometer (Mo Kα radiation: λ = 0.71073 Å). X-ray data analysis was performed with the *SHELXT*(42) and *SHELXL*(43) programs using the *Olex2* interface.^44^ All the hydrogen atoms were refined as “riding”. The thermal displacement parameters of the non-hydrogen atoms were refined anisotropically. The contribution of the disordered solvent was removed using the *SQUEEZE* option of *PLATON* operated with the *Olex2* interface.^44^ The estimated total solvent-accessible void space (1.2 Å probe) was 1141.5 Å^3^ per the unit cell (30.0%) and 65 electrons per unit cell. The number of squeeze electrons correlates with the electron density of 0.9DMF and 3.1H_2_O molecules per unit cell (67 electrons). The CCDC number is 2335633.

### Magnetic Measurements

The dc magnetic susceptibilities of the solvated and desolvated **2**–**4** were measured on a Quantum Design MPMS-XL7AC SQUID magnetometer equipped with a 7 T coil under a static field of 0.5 T. The solvated samples were measured during heating in the range of 10–350 K. The desolvated samples were measured during cooling (400–10 K) and then heating (10–400 K). These desolvated samples were used after the PXRD measurement, in which the sample was heated to 473 K. In addition, the initial temperature was 400 K, to eliminate the captured guest molecules when the PXRD measurements were cooled to room temperature (RT). The magnetic data were corrected using separately measured diamagnetic blank data from the sample holder. The diamagnetic contribution of the sample itself was estimated from Pascal’s constant.^45^

## Results and Discussion

### Synthesis and Characterization

Complexes **2**–**4**·solv were prepared by the method for **1**·solv^34^ with slight modifications. The structure of **4**·solv was confirmed by the SCXRD analysis (see below for details). However, **2** and **3** were not identified by SCXRD because the Fe/Co ratios could not be determined. The crystal structures of **2** and **3** were confirmed by PXRD measurements, which showed that the solvated forms of these compounds are isostructural with **1** and **4**. The exact *x* ratios in mixed Co–Fe crystals **2** and **3** were determined by ICP–MS and further confirmed by EDS. The presence of crystal solvents in **2**–**4**·solv was verified by elemental and thermal analyses.

### Single-Crystal X-ray Crystallography

**Figure 1:**
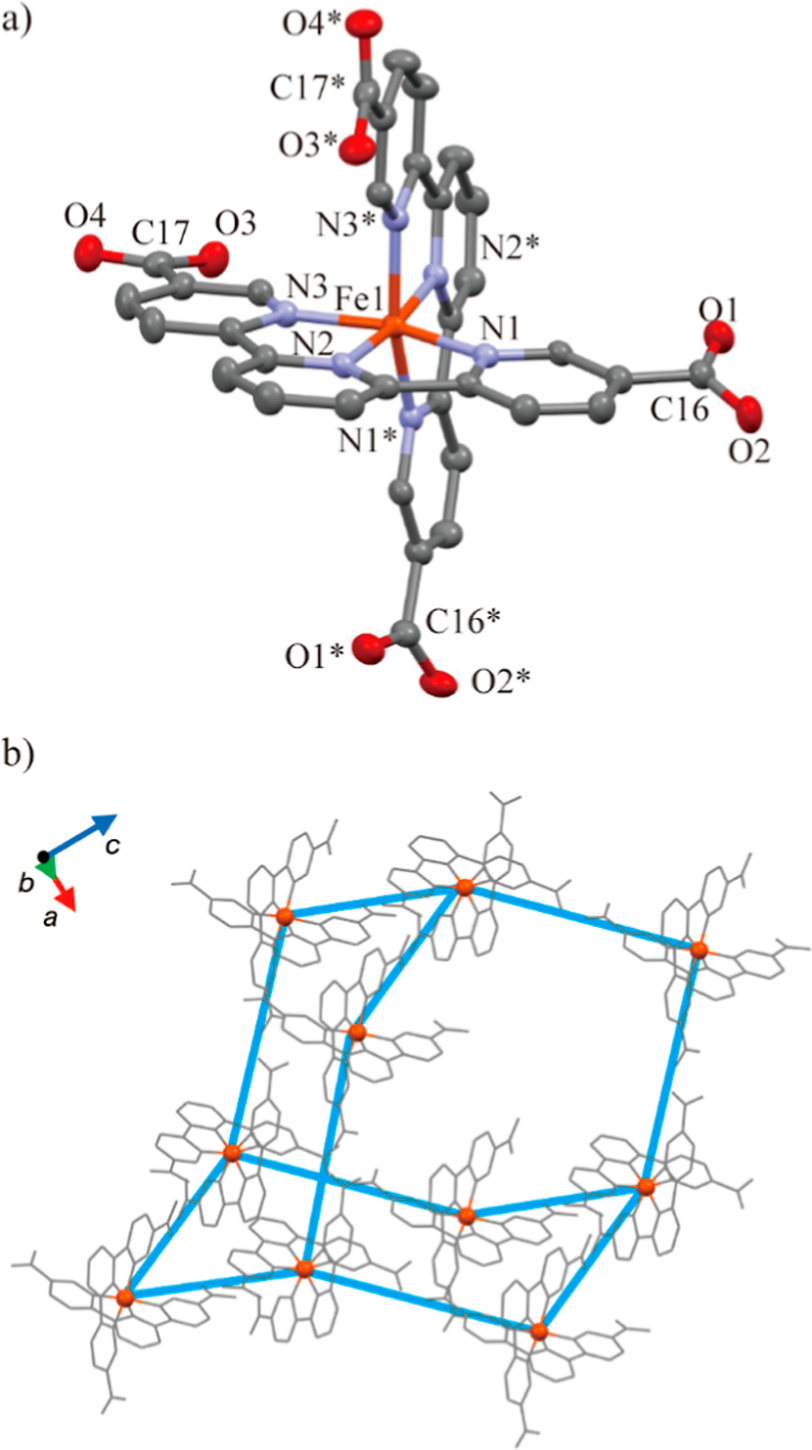
(a) Molecular structure of **4**·solv. The thermal ellipsoids for non-hydrogen atoms are drawn at 50%. Symmetry code: * = 1/2 – *x*, 1/2 – *y*, *z*. (b). H-bonded diamond network. The orange and gray colors represent Fe and nonmetal atoms, respectively. The blue lines represent the connection of the Fe centers through the H-bonds. The H atoms are omitted for clarity.

Complex **4**·solv (*x* = 0) crystallized in a tetragonal *P*4_2_/*n* space group, as shown in Table 1 and Figure 1a, which is isomorphic to **1**·solv (*x* = 1).^34^ The cell parameters *a* and *V* of **4**·solv are larger +0.43% and +0.10% than those of **1**·solv, while the cell parameter *c* of **4**·solv is smaller −0.77%. The Fe^2+^ center is shown to be 6-coordinated with two tpy moieties, giving the N_6_ environment. The structural parameters around the metal centers are summarized in Table 2. The lengths of Fe1–N1, Fe1–N2, and Fe1–N3 are 1.9861(13), 1.8932(13), and 1.9765(13) Å, respectively. The N1 and N3 atoms are in an equatorial (eq) position, while the N2 atom is in an axial (ax) position. The mean of the M–N lengths (*d*_mean_) is 1.9519(13) Å (M = Fe), which is −7.0% shorter than that of 2.0989(11) Å for **1**·solv (M = Co). The coordination environments for **1**·solv and **4**·solv around the metal center are assigned to the octahedral (*O*_h_) geometry using by the *SHAPE* software.^46^ The distortion parameters (Σ and Θ)^47^ to the *O*_h_ for **4**·solv are estimated to be 80.85° and 305.7°, respectively. This result indicates the suppressed *O*_h_ geometry along the axial (N2–Fe1–N2*) direction. In addition, the structural parameters reproduce to the typical values for the *ls*-Fe^2+^ complexes with the N_6_ environment.^40^

**Table 1:** Selected Crystallographic Data for **4**·solv and **1**·solv as the Reference

	4·solv	1·solv^a^
formula	C_34_H_20_FeN_6_O_8_	C_34_H_20_CoN_6_O_8_
Fw	696.41	699.49
*T*/K	90	93
crystal system	tetragonal	tetragonal
space group	*P*4_2_/*n*	*P*4_2_/*n*
*a*/Å	14.4439(4)	14.3814(2)
*c*/Å	18.2120(5)	18.3531(4)
*V*/Å^3^	3799.5(2)	3795.87(13)
*Z*	4	4
*d*_calcd_/g cm^–3^	1.217	1.224
μ(Mo Kα)/mm^–1^	0.450	0.505
*R*(*F*)^b^(*I* > 2σ(*I*))	0.0418	0.0311
*R*_w_(*F*^2^)^c^ (all data)	0.1242	0.0807
goodness of fit	1.049	1.050
no. unique reflns	5386	4512

a Ref (34).

b R = Σ||*F*_o_| – |*F*_c_||/Σ|*F*_o_|.

c *R*_*w*_ = [Σ*w*(|*F*_o_| – |*F*_c_|)^2^/Σ*w*|*F*_o_|^2^]^1/2^.

**Table 2:** Selected Geometry Parameters for **4**·solv and **1**·solv as the Reference

	4·solv	1·solv^a^
M–N1 (eq)/Å	1.9861(13)	2.1326(11)
M–N2 (ax)/Å	1.8932(13)	2.0327(10)
M–N3 (eq)/Å	1.9765(13)	2.1313(11)
*d*_mean_/Å	1.952	2.099
octahedron (*O*_h_)^b^	2.008	4.254
trigonal prism (*D*_3h_)^b^	10.624	8.655
Σ/deg	80.85	119.53
Θ/deg	305.69	461.84

a Ref (34).

b *SHAPE* software.^46^

Four C–O bond lengths at the carboxy/carboxylate sites in **4**·solv (C16–O1, C16–O2, C17–O3, and C17–O4) are 1.220(2), 1.302(2), 1.276(2), and 1.238(2) Å, respectively. The typical C–O bond lengths are 1.226(20) and 1.305(20) Å in the carboxy group and 1.255(10) Å in the carboxylate group.^48^ Therefore, the C16 and the C17 sites for **4**·solv are assigned to the carboxy and carboxylate groups, respectively. This result agrees with **1**·solv. The intermolecular O2···O3 distance is 2.479(2) Å, which is smaller than the sum of the van der Waals radii (O/O: 3.04 Å).^49^ This value indicates the formation of a single {O–H···O}-type H-bond such as **1**·solv (2.4782(13) Å).

The H-bonds formed the diamond-typed iron framework, as shown in Figure 1b. In addition, the H-bonded diamond frameworks exhibited a 4-fold interpenetrating situation with two 1-D pores along the [110] and [110̅] directions. The void spaces were occupied by the disordered crystal solvents, which were considered in the *SQUEEZE*/*PLATON* program. From the calculations, the solvent-accessible void volume was estimated to be 30.0% per unit cell. An electron count of 65 electrons per formula unit was obtained. This electron count is close to the 0.9DMF (36 electrons) and 3.1H_2_O (31 electrons) solvents, which are estimated by the elemental analysis.

The void volume of **4**·solv is slightly larger than that of **1**·solv (27.9%), with the *V* parameter of **4**·solv also exceeding that of **1**·solv. Considering the relatively short *d*_mean_ value and the comparable distance of H-bonds in **4**·solv compared to **1**·solv, the difference in the distortion of the *O*_h_ geometry around the metal center may be a crucial factor influencing the void space within the crystal structure. Removal of the octahedral distortion causes the angle between the two tpy moieties to approach a right angle. Specifically, the dihedral angle of **4**·solv is 85.19°, slightly larger than that of 83.46° for **1**·solv. These dihedral angles play a role in determining the angle formed by lattice points in the diamond framework. The H-bonded diamond framework of **4**·solv adopts a chair configuration with the Fe···Fe distance of 13.6833(2) Å, with four angles of 116.29° and two angles of 96.56°. These angles are defined by three metal centers in adjacent complex units. In contrast, the chair configuration of **1**·solv has the distance of 13.6975(2) Å with the four 116.67° and two 95.87° angles. Although the Fe···Fe distance of **4**·solv is slightly smaller than that of **1**·solv, the angles of **4**·solv are close to those of typical diamond/carbon (109.5°) compared to **1**·solv. Consequently, the suppression of distortion of the diamond framework for **4**·solv contributes to an increase in the cell volume and void space.

### Thermal Analysis

**Figure 2:**
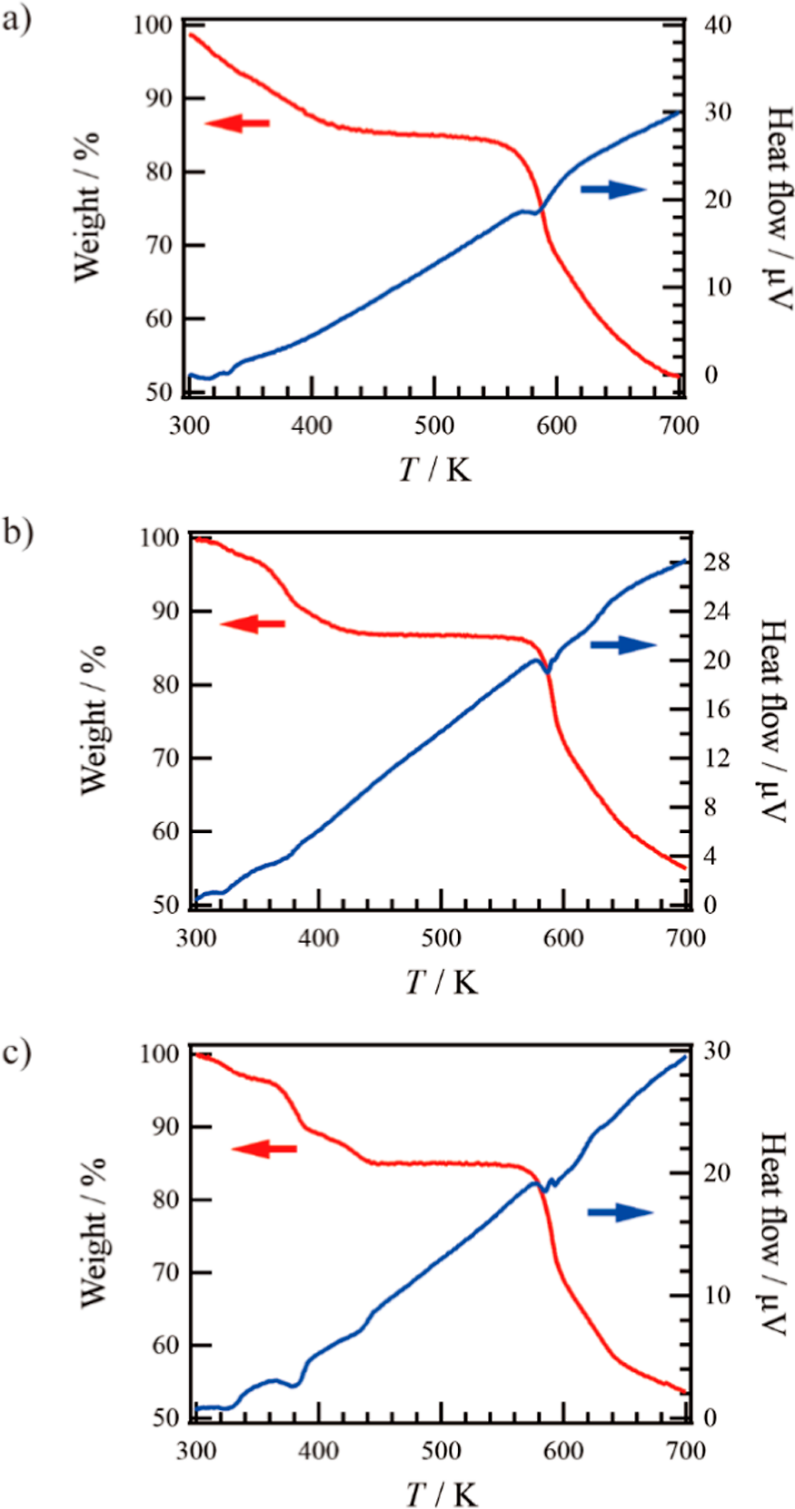
TG (red) and DTA (blue) results for (a) **4**·solv (*x* = 0), (b) **3**·solv (*x* = 0.55), and (c) **2**·solv (*x* = 0.88) in 300–700 K.

Compound **4**·solv (*x* = 0) was evaluated by TG and DTA techniques as shown in Figure 2a. The TG curve showed a gradually decreasing process up to 400 K, indicating that it is difficult to determine which solvent, H_2_O or DMF, is responsible for the observed desorption. The weight loss (Δ*w*) for the process was about −14.9%. This result indicates the desorption of crystal solvents. Upon further heating, the TG curve showed a plateau up to 560 K. This plateau implies that **4**·solv was completely desolvated (**4**·desolv, fw: 696.45). The Δ*w* is equivalent to fw 121.9, which corresponds to the sum of the 0.9DMF (fw 65.80) and 3.1H_2_O (fw 55.86) solvents observed in the results of the elemental and crystallographic analyses.

Compound **3**·solv (*x* = 0.55) was analyzed by TG and DTA techniques (Figure 2b). The TG curve showed a gradual two-step reduction process up to 450 K, followed by a plateau up to 550 K. This reduction process is similar to that observed for **4**. The total Δ*w* of **3** up to 450 K was about −13.2%. Based on the fully desolvated molecular weight of **3** (fw 698.14), the Δ*w* value is equivalent to fw 105.4, which corresponds to the sum of the solvents (0.9DMF and 2.2H_2_O, fw 106.2). These values agree with the elemental analysis results. The two-step desorption process can be further subdivided into weight losses of about −2.3% and −10.9% for the first and second steps, respectively. Using the above calculated fw of 106.2, these values correspond to fw of 18.50 and 87.70, respectively. In the first step, it is assumed that the H_2_O molecules (47%) are gradually desorbed owing to the smaller molecular size of H_2_O compared to DMF, which facilitates its desorption. In the second step, all DMF molecules are gradually desorbed along with the remaining H_2_O molecules (53%).

Compound **2**·solv (*x* = 0.88) was analyzed by TG and DTA techniques (Figure 2c). The TG curve showed a three-step reduction process up to 450 K and then a plateau up to 550 K. This reduction process of **2** is similar to that observed for **1**.^34^ The total Δ*w* of **2** up to 450 K was about −15.1%. Considering the fully desolvated molecular weight of **2** (fw 699.16), the Δ*w* value is equivalent to fw 123.7, which corresponds to the sum of the solvents, 1.1DMF and 2.4H_2_O (fw 124.4). These results agree with the elemental analysis results. The three-step desorption process can be broken down into weight losses of about −3.6%, −6.9%, and −4.7% for the first, second, and third steps, respectively. Based on the above calculated fw 123.7, these values correspond to fw of 28.67, 56.53, and 38.50, respectively. Note that the second step shows a more abrupt decrease than the other two steps. From these observations, it is assumed that in the first step, the H_2_O molecules (66%) are gradually desorbed. In the second step, the DMF molecules (70%), which have a relatively large molecular size, begin to be desorbed. Finally, in the third step, the remaining H_2_O molecules (34%) are desorbed together with the DMF (30%).

### Powder X-ray Diffraction

**Figure 3:**
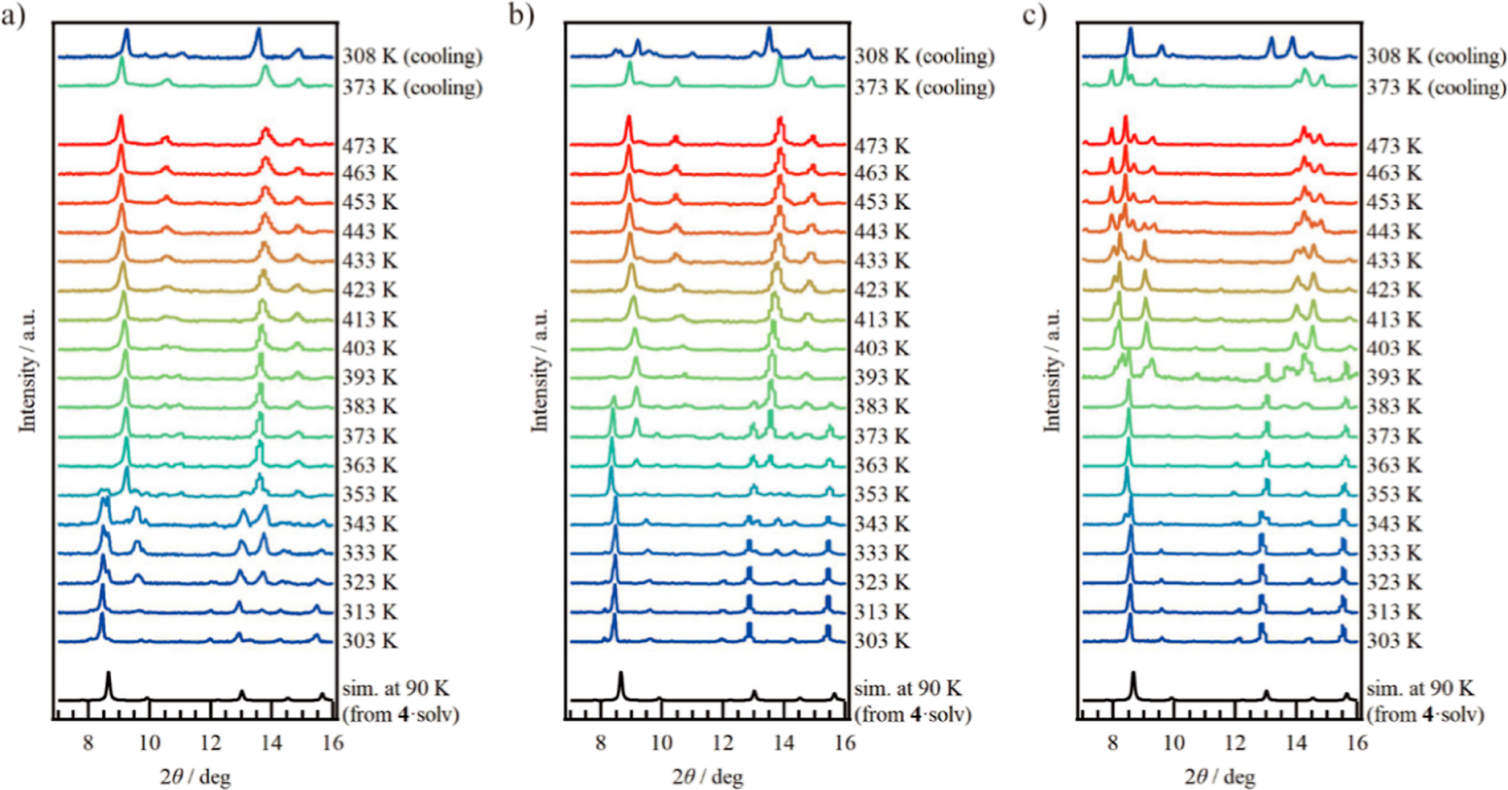
Variable-temperature PXRD measurements of (a) **4**·solv, (b) **3**·solv, and (c) **2**·solv during the heating and cooling processes. The simulation result (black line) was derived from the SCXRD result for **4**·solv measured at 90 K.

Variable-temperature powder X-ray diffraction (VT-PXRD) was applied to **4**·solv (*x* = 0) as shown in Figure 3a. The simulation pattern from the above SCXRD analysis has been superimposed on the figure. The PXRD pattern at 303 K agrees well with the simulation pattern. Upon heating, the PXRD patterns changed from 323 to 353 K, which is associated with the desorption of the crystal solvents observed in the thermal analysis results. Upon further heating from 363 to 473 K, the peak at about 9° was shifted to the lower angle, indicating thermal expansion. During the cooling process from 473 K, the PXRD patterns showed no change down to 373 K. The spectrum at 308 K changed slightly and is close to the spectra at 363–403 K during the heating process. The result suggests that the H_2_O molecules were captured from the ambient environment during the cooling process. Compared to **1**·solv, the structural changes due to solvent desorption in **4**·solv were small. The increase in the void space allows the crystalline solvents to be more accessible without affecting the structure.

The VT-PXRD result for **3**·solv (*x* = 0.55) is shown in Figure 3b. The PXRD pattern for **3**·solv at 303 K is consistent with the simulation pattern derived from **4**·solv at 90 K, and the structural change during the heating process is also similar to that of **4**·solv. Upon heating, the PXRD patterns changed between 363 and 383 K, corresponding to the second desorption step of the crystal solvents observed in the thermal analysis. With further heating, the peak at about 9° shifted to a lower angle, indicating thermal expansion. In contrast, upon cooling, the spectrum at 308 K showed slight changes, suggesting that the H_2_O molecules were captured from the ambient environment.

The VT-PXRD result for **2**·solv (*x* = 0.88) is shown in Figure 3c. The PXRD pattern for **2**·solv at 303 K matches the simulation pattern derived from **4**·solv at 90 K, similar to the case of **3**·solv. However, the structural changes upon heating are more comparable to those of **1**·solv (Figure S6). Upon heating, the PXRD patterns changed at 333, 390, and 423 K, which is agreement with the results of thermal analysis. Notably, the second desorption step, which likely corresponds to the removal of DMF, caused significant changes in the PXRD patterns, consistent with the behavior for **1**·solv (Figure S6). Due to the similar Co ion ratios between **2** and **1**, their crystal structures are likely to be analogous. In fact, upon cooling from 473 to 308 K, the PXRD pattern for **2** is reproduces that of **1**.

### Magnetic Properties

**Figure 4:**
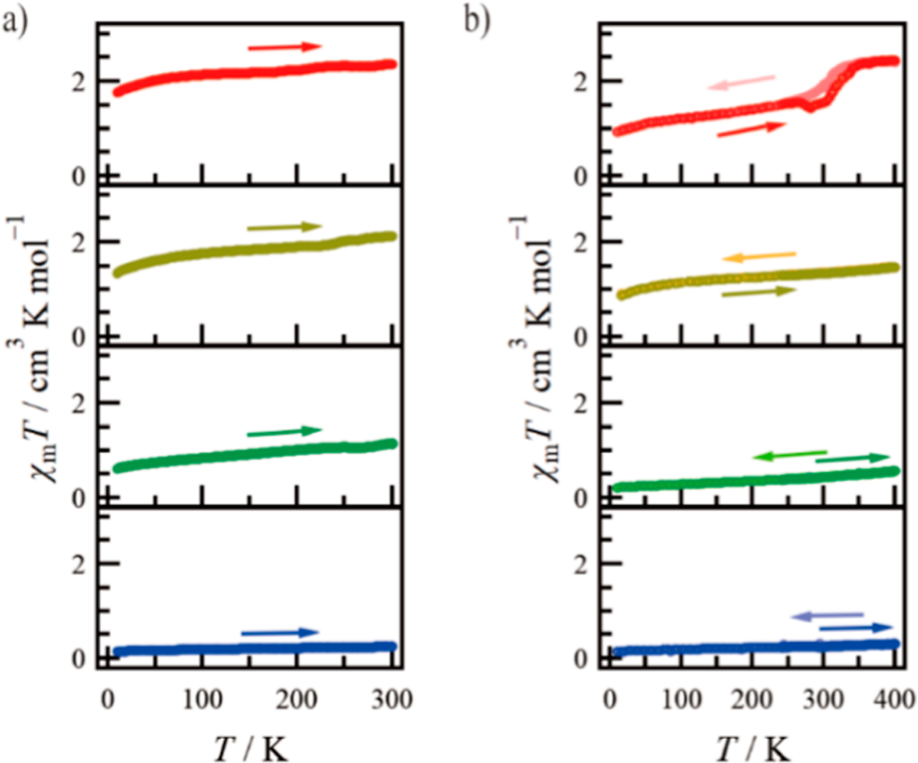
Temperature dependence of the product χ_m_*T* for the (a) solvated and (b) desolvated forms of **1** (red), **2** (yellow), **3** (green), and **4** (blue). (b) The filled (light-colored) and open (deep-colored) circles represent the cooling and heating processes, respectively.

The temperature dependence of χ_m_*T* for **4**·solv (*x* = 0) was measured at 0.5 T in 10–300 K, as shown in Figure 4a (blue circle). The χ_m_*T* value of **4**·solv was 0.253 cm^3^ K mol^–1^ at 300 K. The *ls*-Fe^2+^ center (*S* = 0) is expected to show the χ_m_*T* value of zero. The residual χ_m_*T* value for **4**·solv indicates the presence of the *hs*-Fe^3+^ center due to the air oxidation of the Fe^2+^ ions. During the cooling process down to 10 K, the χ_m_*T* value gradually decreased. The χ_m_*T* plots for **4**·desolv illustrating the cooling and then heating processes in 10–400 K are shown in Figure 4b (blue filled and open circles, respectively). The χ_m_*T* value at 400 K was 0.313 cm^3^ K mol^–1^ and gradually decreased on cooling to 10 K. This behavior followed the same path during the heating process.

The temperature dependence of χ_m_*T* for **3**·solv (*x* = 0.55) was measured at 0.5 T in 10–300 K, as shown in Figure 4a (green circle). The χ_m_*T* value of **3**·solv was 1.154 cm^3^ K mol^–1^ at 300 K. Considering the presence of the iron(III) impurities observed in **4**, the estimated value based on the single Co^2+^ center is 1.032 cm^3^ K mol^–1^ at 300 K. Next, if the *g* values in the *hs*- and *ls*-Co^2+^ centers are the same as in **1**·solv (*g* = 2.25),^34^ the expected χ_m_*T* values are 2.373 and 0.474 cm^3^ K mol^–1^, respectively. Compound **3** has the Co ions with *x* = 0.55, and based on the χ_m_*T* value at 300 K, the spin state for the Co^2+^ center in **3**·solv is estimated to be 74.6% in the hs state and 25.4% in the ls state. The value in the whole molecule (including the Fe center) is also estimated to be the hs ratio (γ_*hs*_) = 41.0%. Upon cooling, the χ_m_*T* value showed a gradual decrease, reproducing the same behavior as **1**. The χ_m_*T* plots for **3**·desolv, illustrating the cooling and then heating processes in 10–400 K, are shown in Figure 4b (green filled and open circles, respectively). The χ_m_*T* value at 400 K was 0.568 cm^3^ K mol^–1^ and gradually decreased on cooling to 10 K. This behavior followed the same path during the heating process. The estimated value based on the single Co^2+^ center is 0.418 cm^3^ K mol^–1^ at 400 K. If the *g* values in the *hs*- and *ls*-Co^2+^ centers are the same as **1**·desolv (*g* = 2.22),^34^ the expected χ_m_*T* values are 2.310 and 0.462 cm^3^ K mol^–1^, respectively. By means of the above calculation approach for the solvated state, the Co^2+^ center in **3**·desolv shows the hs state of 17.1% (γ_*hs*_: 9.39%). The small γ_*hs*_ value of **3** indicates the strong effect of the inclusion of the Fe^2+^ center.

The temperature dependence of χ_m_*T* for **2**·solv was measured at 0.5 T in 10–300 K, as shown in Figure 4a (yellow circle). The χ_m_*T* value of **2**·solv was 2.118 cm^3^ K mol^–1^ at 300 K. Likewise **3**, considering the iron(III) impurities observed in **4**, the estimated value based on the single Co^2+^ center is 2.090 cm^3^ K mol^–1^ at 300 K. The Co^2+^ center in **2**·solv exhibits the hs state of 99.9% (γ_*hs*_: 87.9%). The χ_m_*T* plots for **2**·desolv illustrating the cooling and then heating processes in 10–400 K are shown in Figure 4b (yellow filled and open circles, respectively). The χ_m_*T* value at 400 K was 1.472 cm^3^ K mol^–1^ and gradually decreased on cooling to 10 K. This behavior followed the same path during the heating process. The estimated value based on the single Co^2+^ center is 1.437 cm^3^ K mol^–1^. The Co^2+^ center in **2**·desolv shows the hs state of 63.2% (γ_*hs*_: 55.6%). The stabilization of the *ls* state due to the metal-dilution effect is shown as well as the case of **3**.

**Figure 5:**
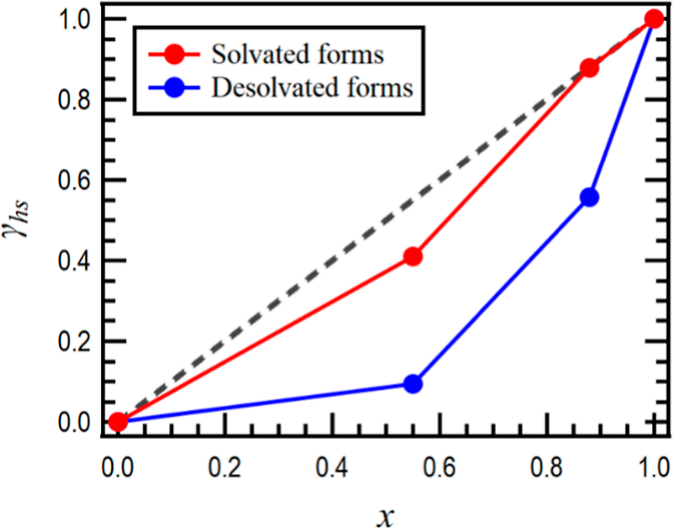
Concentration of the Co^2+^ center (*x*) dependence of the γ_*hs*_ values for the (red) solvated and (blue) desolvated forms. The dashed black line represents the maximum of the γ_*hs*_ value at each concentration.

Figure 5 illustrates the concentration dependence of the γ_*hs*_ values, with red and blue markers representing the solvated and desolvated states, respectively. The dashed black line indicates the maximum γ_*hs*_ values for each concentration. The solvated state results (red markers) are aligned with the dashed black line. This suggests that the introduction of Fe^2+^ ions has a minimal effect on the spin state of the *hs*-Co^2+^ center in the solvated state. The presence of solvents plays a crucial role in determining the framework and the coordination environment around the Co^2+^ center, exerting a greater influence than the metal-dilution effect. In contrast, the desolvated state shows a significant decrease in the γ_*hs*_ values with a slight increase in the Fe^2+^ ratio, a trend different from that observed in the solvated state. The transition of Co^2+^ to the *ls* state upon the introduction of *ls*-Fe^2+^ can be attributed to the similarity in their ionic radii (*ls*-Co^2+^, 0.65 Å; *ls*-Fe^2+^, 0.61 Å).^41^

The difference in the metal-dilution effect observed between the solvated and desolvated states is attributed to differences in their crystal structures. The structure of the solvated state has been described in the above SCXRD studies. Specifically, the solvated state is characterized by 1-D pores that extend in two directions and encapsulate solvents (DMF and water molecules). The relatively large molecular size of DMF could play a crucial role in stabilizing the structure of the solvated state. Forming a flexible framework capable of solvents requires not only the selection of the appropriate structural network motif but also the incorporation of the hs state, which enhances flexibility in coordination bonds within the complex unit. Note that this spin-state selection for the Co^2+^ ion is feasible because it exhibits a smaller energy gap between the hs and *ls* states compared to the Fe^2+^ ion. In contrast, the Fe^2+^ ion does not exhibit a significant solvent effect sufficient to override the ligand field, as demonstrated by the results for **4**. These results indicate that in the solvated state, the spin state of Co^2+^ ions is more strongly influenced by the presence of solvent molecules, which play a dominant role in determining the crystal structure, than by the introduction of nonmagnetic *ls*-Fe^2+^ ions with a similar ionic radius.

In the desolvated state, although the exact crystal structure remains unclear, the removal of solvent is thought to remove factors that promote the *hs*-Co^2+^ state. Magnetic measurements on the desolvated state of **1** revealed ST behavior, indicating an enhanced ligand field. In this environment, the metal-dilution effect, commonly observed in spin crossover (SCO) studies of Fe^2+^ complexes, becomes apparent. For the Fe^2+^ complexes, nonmagnetic Zn^2+^ ions (the inactive metal center) are typically used as dilution sources. Reducing the fraction of Fe^2+^ ions suppresses cooperative phenomena such as hysteresis. However, the ST temperature remains relatively insensitive to the amount of doping,^35−39^ as it is primarily determined by the ligand field strength at the spin sites. On the other hand, an interesting observation in this study is that a small doping of *ls*-Fe^2+^ ions (the inactive metal center) into the Co^2+^ center (the active metal center) suppresses the ST phenomena. The compounds in this study exhibit the H-bonded structure, which could facilitate the propagation of structural changes more effectively than covalently bonded MOFs. This feature enhances the cooperative effect in the ST behavior. In fact, the ST behavior of Co^2+^ in **1** shows the abrupt transition accompanied by thermal hysteresis, indicating strong cooperativity. The pronounced metal-dilution effect observed in the desolvated states can be attributed to the combination of the high cooperativity of the structure and the use of Co^2+^ metal center. These factors amplify the effect of even small amounts of *ls*-Fe^2+^ doping, resulting in significant changes in the ST behavior.

## Conclusion

In this study, to investigate the metal-dilution effect on the structural and magnetic properties of **1**, the Co/Fe compounds [Co^II^_*x*_Fe^II^_1–*x*_(HL)_2_] (**2**, *x* = 0.88; **3**, *x* = 0.55; **4**, *x* = 0) were prepared. Structural analysis showed that **4** had a larger cell volume and void space than **1**, due to difference in the distortion of the octahedral geometry around the metal center. Thermal and elemental analyses estimated the amount of guest molecules in **1**–**4**·solv, revealing variations in solvent desorption process as a function of Fe^2+^ ratio, as well as changes in void space. Compound **1**·desolv exhibited both normal and reverse ST behavior with the thermal hysteresis loop, while the desolvated forms **2** and **3** showed no ST behavior, highlighting the pronounced metal-dilution effect. In the solvated forms in **1**–**4**, the γ_*hs*_ values closely matched the Co^2+^ ratio, showing no metal-dilution effect. The presence of guest molecules creates the void in the framework of **1**, possibly resulting in a small cooperative effect that masks the metal-dilution effect. However, solvent desorption enhances the cooperative effect, favoring the stabilization of the *ls*-Co^2+^ center in combination with the metal-dilution effect. The results indicate that, in H-MOF, the solvent dependence has a more significant impact on the magnetic properties than the metal-dilution effect due to the flexible framework. Furthermore, the introduction of *ls*-Fe^2+^ in this study led to the disappearance of the ST behavior in desolvated **1** at relatively low incorporation levels. In the future, it is expected that the modulation of magnetic properties, such as the ST temperature and the width of the hysteresis loop, could be achieved by tuning the flexibility of the hydrogen-bonded framework through chemical modification of the ligands, changing the type of guest molecules (e.g., size and polarity), or changing the introduced metal ions.

## Supporting Information Available

IR spectra and SEM results for **2**–**4** and VT-PXRD of **1** (PDF)
2335633 (CIF)

## Supplementary Material

ao4c10686_si_001.pdf

ao4c10686_si_002.cif
