# Supplementary material for: Metal-Dilution Effect on Spin Transition Behavior of Solvated/Desolvated Hydrogen-Bonded Cobalt(II)-Organic Frameworks
Source: ACS Omega. 2025 Jan 16;10(3):3182–9. doi: 10.1021/acsomega.4c10686 (PMC11780442; doi:10.1021/acsomega.4c10686)
Supplement: Supplementary file 1 — ao4c10686_si_001.pdf [file ao4c10686_si_001.pdf]

Metal-Dilution Effect on Spin Transition Behavior of Solvated/Desolvated Hydrogen-Bonded Cobalt(II)-Organic Frameworks

Keisuke Yamato, Takuya Kanetomo* and Masaya Enomoto*

Department of Chemistry, Faculty of Science Division 1,

Tokyo University of Science, 1-3 Kagurazaka, Shinjuku-ku, Tokyo, 162-8603, Japan

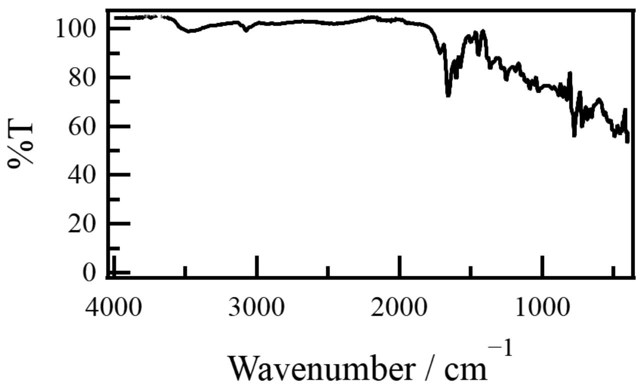

Figure S1. IR spectrum for **2**·solv (x = 0.88).

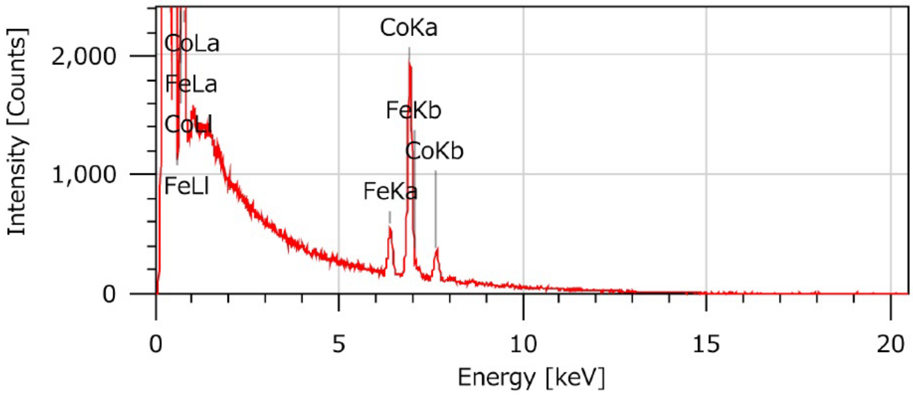

Figure S2. EDS spectrum for **2**·solv (x = 0.88).

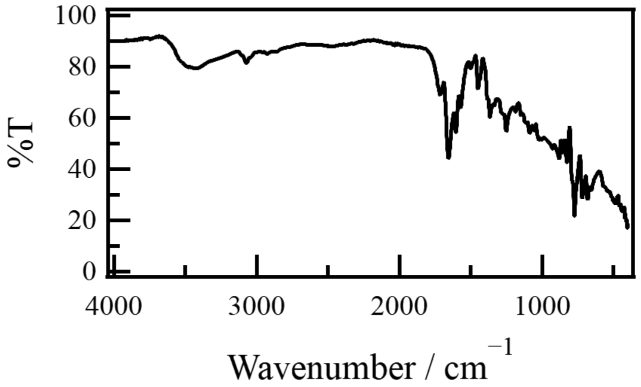

Figure S3. IR spectrum for **3**·solv (x = 0.55).

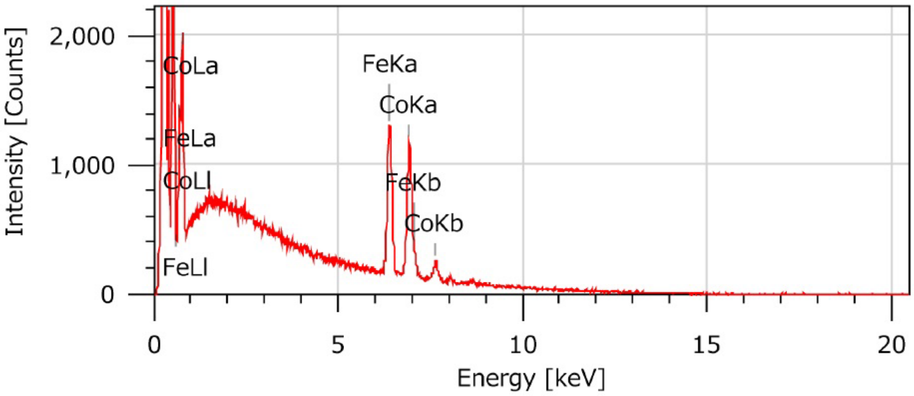

Figure S4. EDS spectrum for **3**·solv (x = 0.55).

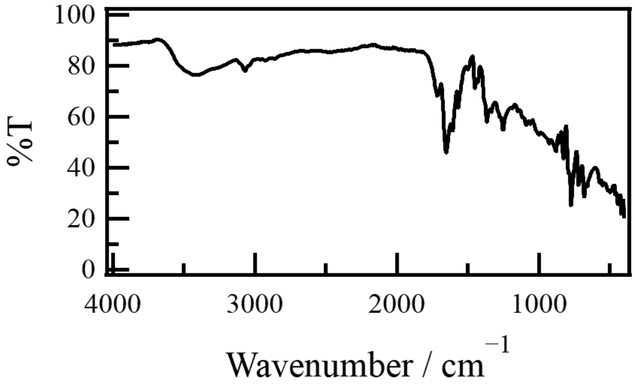

Figure S5. IR spectrum for **4**·solv (x = 0).

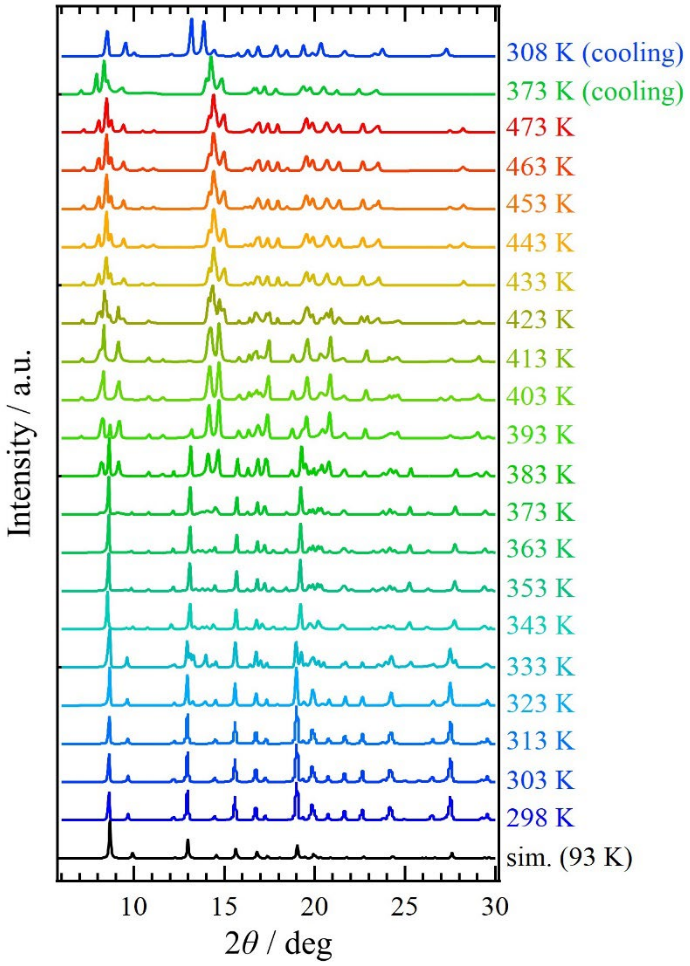

Figure S6. Variable-temperature PXRD measurements of **1**·solv from 298 to 473 K and from 473 to 308 K. The simulation results (black line) were derived from the single-crystal X-ray crystallographic results obtained at 93 K. This graph is described in the reference: Kanetomo, T. et al., *Dalton Trans*. **2022**, *51*, 5034.
